# The association between triglycerides and ectopic fat obesity: An inverted U-shaped curve

**DOI:** 10.1371/journal.pone.0243068

**Published:** 2020-11-30

**Authors:** Yang Zou, Guotai Sheng, Meng Yu, Guobo Xie

**Affiliations:** 1 Medical Department of Graduate School, Nanchang University, Nanchang, Jiangxi Province, China; 2 Department of Cardiology, Jiangxi Provincial People's Hospital Affiliated to Nanchang University, Nanchang, Jiangxi Province, China; Capital Medical University, CHINA

## Abstract

**Background:**

Ectopic fat obesity and triglycerides are risk factors for diabetes and multiple cardiovascular diseases. However, there have been limited studies on the association between triglycerides and ectopic fat obesity. The purpose of this study was to explore the association between triglycerides and ectopic fat obesity.

**Methods and results:**

In this cross-sectional study, we retrospectively analyzed 15464 adult participants recruited by Murakami Memorial Hospital (8430 men and 7034 women, average age of 43.71 ± 8.90). All patients were divided into two groups according to the threshold used to diagnose hypertriglyceridemia. The logistic regression model was used to analyze the association between triglycerides and the risk of ectopic fat obesity, and the generalized additive model was used to identify the nonlinear association. In this study population, the prevalence of ectopic fat obesity was 17.73%. After adjusting other covariables, triglycerides were positively correlated with the risk of ectopic fat obesity (OR: 1.54, 95% CI:1.41–1.69, *P*<0.0001). Through smooth curve fitting, we found that there was an inverted U-shaped curve association between triglycerides and ectopic fat obesity. This association remained unchanged even if the adjusted covariables were removed from the model, and the inflection point of the curve was 3.98. When triglyceride levels were ≤3.98, triglycerides were positively correlated with the risk of ectopic fat obesity (OR:1.784, 95% CI:1.611–1.975, *P*<0.0001). When triglyceride levels were >3.98 (right side of the inflection point), there was a negative correlation (OR:0.519, 95% CI:0.333–0.810, *P* = 0.0039).

**Conclusions:**

Our research showed that there is a significant association between triglycerides and ectopic fat obesity. This relation is not a simple linear relationship but instead an inverted U-shaped curve association.

## Introduction

Obesity is frequently regarded as a collection of oversized and overweight physical features in our daily life. The World Health Organization defines obesity as abnormal or excessive fat accumulation, which may damage health [1]. Adipose tissue is an active metabolic organ, and it participates in physiological activities among various systems. However, excessive fat accumulation adversely affects almost all physiological functions of the human body, and it directly or indirectly increases the risk of hypertension, chronic kidney disease, type 2 diabetes, obstructive sleep apnea and a variety of cardiovascular and cerebrovascular diseases. Excessive fat accumulation even plays an essential role in the pathogenesis of cancer [2–8]. Obesity is gradually causing a severe economic and disease burden to the world [2,8]. Since the 1980s, the global prevalence of overweight and obesity has doubled in more than 70 countries, and nearly one-third of the world's population is classified as overweight or obese [2]. Notably, obesity has been regarded as a body surface characteristic in the past, but now it is considered to be a complex disease with multiple causes, which have been focused on by more and more people [9].

Over the past few decades, obesity has been mainly assessed based on body mass index (BMI). Currently, many people oppose the use of a single index of BMI in the diagnosis of obesity because the sensitivity of BMI is and there is a vast difference in the ratio of fat among individuals. Thus, relying solely on BMI to evaluate obesity may hinder future interventions [2]. Accurate assessment of obesity is necessary, and under the current trend of the obesity pandemic, this work contains more practical significance. Recently, a series of studies based on obesity phenotype have focused on potential phenotypes, namely, "visceral fat obesity" and "ectopic fat obesity" [2,10–12]. With regard to ectopic fat, it is defined as extra adipose tissue that appears in locations unrelated to the initial storage of adipose tissue, such as fat storage in the liver and muscle, pericardial fat, perivascular fat and perirenal fat, and liver fat is representative of ectopic fat accumulation [13,14]. Ectopic fat obesity has been closely related to dyslipidemia, diabetes and cardiovascular disease in previous studies [7,15–17]. The accumulation of triglycerides (TGs) in different tissues is an essential risk factor for diabetes and cardiovascular disease [18–21]. To date, there have only been a few studies on the association between TGs and the risk of ectopic fat obesity [22–24], and the guidelines for the management of blood lipids in patients with ectopic fat obesity are not clear. Ectopic fat obesity is a massive health problem that has not received much attention. Therefore, it is imperative to explore and intervene with the risk factors of ectopic fat obesity.

## Methods

### Research population and design

This study was a cross-sectional study designed to evaluate the association between TGs and ectopic fat obesity. The clinical data of our study population was from a public database (https://datadryad.org, doi.org/10.5061/dryad.8q0p192), provided by Okamura et al. [16]. In this study, all participants were at least 18 years old, and clinical data were extracted for subjects who participated in the physical examination program at Murakami Memorial Hospital from 2004 and 2015. Through this database, we investigated the risk of TGs and ectopic fat obesity. The personal information of the participants was deleted and replaced by a health examination number. Research ethical approval and informed consent from the patients were obtained in previous studies [16], indicating that this study did not require ethical research approval.

### Data collection

The baseline data of all populations were obtained by standardized self-administered questionnaires, including smoking/drinking habits, body weight, height, sex, age, waist circumference (WC) and habit of exercise. To measure biochemical blood indicators after an overnight fast, venous blood was drawn for testing of the following indicators: alanine aminotransferase (ALT), aspartate aminotransferase (AST), gamma-glutamyl transferase (GGT), total cholesterol (TC), hemoglobin A1c (HbA1c), fasting blood glucose (FPG), TG and HDL cholesterol (HDL-C). The concentrations of TG were determined using a MODULAR ANALYTICS automatic analyzer (HITACHI Hitechnologies Co., Ltd., Tokyo, Japan). In this observational study, cases with the following characteristics were excluded: (1) participants who had heavy drinking habits or diagnosis of alcoholic fatty liver disease [25]; (2) participants diagnosed with viral hepatitis B or C; (3) participants who took any drug and who had diabetes at the baseline examination; (4) participants with missing covariable data; and (5) participants with FPG ≥6.1 mmol/L.

### Definition

Alcohol status was defined as follows: none or very light drinking, <40 g/week; light drinking, 40–140 g/week; moderate drinking, 140–280 g/week; or heavy drinking, >280 g/week. Smoking status was defined as follows: nonsmokers were defined as participants who never smoked; past smokers were defined as participants who used to smoke but quit before the baseline visit, and current smokers were defined as participants who smoked during the baseline visit. Furthermore, the habit of exercise was defined as participants who participated in any type of exercise more than once a week.

Ectopic fat obesity was defined as fatty liver confirmed by abdominal ultrasound, and trained technicians and experienced doctors made the diagnosis of fatty liver by examining the results of abdominal ultrasonography based on the scores of the following four ultrasound examinations: hepatorenal echo contrast, liver brightness, deep attenuation and vascular blurring [26].

### Statistical analysis

To better understand the association between TGs and ectopic fat obesity, we stratified the study population based on the threshold used to diagnose hypertriglyceridemia (≤1.7 and >1.7). The Kolmogorov-Smirnov test and QQ plots were used to check the normality of distribution of the continuous variables. Normally distributed continuous variables were expressed by mean ± standard deviation, and continuous variables with a skewed distribution were expressed by median (interquartile range). Qualitative variables were described by n or %. To determine differences among the groups, a t-test was used for normally distributed continuous variables, and the Kruskal-Wallis H test was used for continuous variables with a skewed distribution. Qualitative variables were analyzed by the χ^2^ test. Univariate analysis was performed on all variables to assess the risk of ectopic fat obesity initially, and multiple linear regression was used to test the collinearity between variables. According to the variance inflation factor (VIF) [27], the variables with VIF>5 were considered to have severe multicollinearity, and the multivariate logical regression model was used to calculate the correlation between TGs and ectopic fat obesity and evaluate the risk degree. Odds ratios (OR) with 95% confidence intervals (CI) were recorded. Based on the STROBE statement [28], the results of the unadjusted analysis (crude model), fine-tuning adjustment analysis (model I), and the full adjustment analysis (model II) are shown. In addition, we used the generalized additive model (GAM, Restricted Cubic Spline Functions) to identify whether there was a nonlinear association between TG and ectopic fat obesity. When the result was a nonlinear correlation, the inflection point of the curve was identified by Engauge Digitizer software (https://github.com/markummitchell/engauge-digitizer/tree/v11.1), and the two-stage logistic regression model was used to calculate the saturation effect of TG on the occurrence of ectopic fat obesity according to the smoothing curve. On the other hand, in order to explore the possible influencing factors in the risk of TGs and ectopic fat obesity, we conducted stratified analysis and interaction tests in pre-defined subgroups (Stratification of sex, age and BMI according to clinical entry point). The logistic regression model was used to analyze each hierarchical variable, and the likelihood ratio was used to test the modification and interaction of subgroups. Additional, to control for Type I errors across the subgroup analyses, we used the Bonferroni correction (The way of Bonferroni correction is β = α/n, n = number of tests, in this study, using 0.05/3 = 0.0167 as a corrected significance threshold, given the 3 subgroups). Statistical analyses were performed using the R-project 3.4.3 and Empower (R) software packages (www.empowerstats.com; X&Y Solutions Inc.).

## Results

### Study population baseline characteristics

In this study, a total of 20944 participants were recruited, including 12498 men and 8446 women, and 5480 participants who did not meet the inclusion criteria were excluded as follows: 863 participants lacked covariant data; 416 participants had hepatitis B or C virus; 739 participants had heavy drinking habits; 2321 participants took drugs at baseline; 323 participants had diabetes; 808 participants had baseline FPG >6.1 mmol/L, and 10 participants did not participate in the study for unknown reasons. Finally, we evaluated 15464 people who met the inclusion criteria (8430 men and 7034 women with an average age of 43.71 ± 8.90), including 2741 patients (17.73%) with ectopic fat obesity. Tables 1 and 2 summarize the clinical baseline characteristics of the study population. Participants in the hypertriglyceridemia group (>1.7) generally had higher age, BMI, body weight, WC, ALT, AST, GGT, TC, HbA1c, FPG, SBP, DBP and prevalence of ectopic fat obesity compared to the normal TG group (≤1.7). In contrast, individuals in groups with normal TG levels exercised more and had higher HDL-C levels (*P*<0.05). Similarly, individuals with ectopic fat obesity were older and had higher BMI, body weight, WC, ALT, AST, GGT, TC, TG, HbA1c, FPG and blood pressure. In addition, the prevalence of ectopic fat obesity in men was higher than that in women (*P*<0.05).

**Table 1:** Baseline characteristics of participants with or without hypertriglyceridemia (N = 15464).

Variables	TG (mmol/L)		*P*-value
	≤1.7	>1.7	
No. of participants	13992	1472	
Sex, (men)	7132 (50.97%)	1298 (88.18%)	<0.001
Age, (years)	43.54±8.94	45.27±8.37	<0.001
BMI (kg/m^2^)	21.53 (19.73,23.57)	24.38 (22.68,26.36)	<0.001
Body weight (kg)	58.40 (51.10–66.70)	69.65 (63.20–77.30)	<0.001
WC (cm)	75.00 (69.00–81.30)	84.00 (79.50–89.30)	<0.001
Ectopic fat obesity	1960 (14.01%)	781 (53.06%)	<0.001
Habit of exercise	2492 (17.81%)	217 (14.74%)	0.003
Drinking status			<0.001
None	10846 (77.52%)	959 (65.15%)	
Light	1562 (11.16%)	196 (13.32%)	
Moderate	1158 (8.28%)	202 (13.72%)	
Heavy	426 (3.04%)	115 (7.81%)	
Smoking status			<0.001
Never	8501 (60.76%)	530 (36.01%)	
Past	2585 (18.47%)	367 (24.93%)	
Current	2906 (20.77%)	575 (39.06%)	
ALT (IU/L)	16.00 (12.00–22.00)	26.00 (19.00–36.00)	<0.001
AST (IU/L)	17.00 (14.00–21.00)	21.00 (17.00–26.00)	<0.001
GGT (IU/L)	14.00 (11.00–21.00)	26.00 (19.00–40.00)	<0.001
HDL-C (mmol/L)	1.45 (1.22–1.73)	1.05 (0.91–1.22)	<0.001
TC (mmol/L)	5.02 (4.47–5.61)	5.66 (5.12–6.28)	<0.001
TG (mmol/L)	0.69 (0.47–0.98)	2.16 (1.87–2.65)	<0.001
HbA1c (%)	5.15 (4.94–5.40)	5.20 (5.00–5.50)	<0.001
FPG (mmol/L)	5.14±0.41	5.40±0.37	<0.001
SBP (mmHg)	112.50(103.00,123.00)	122.50(112.88,132.50)	<0.001
DBP (mmHg)	70.00 (63.50–77.50)	77.50 (71.00–84.50)	<0.001

Values are n (%) or mean ± SD. Abbreviations: BMI: Body mass index, WC: Waist circumference, ALT: Alanine aminotransferase, AST: Aspartate aminotransferase, GGT: Gamma-glutamyl transferase, HDL-C: High-density lipoprotein cholesterol, TC: Total cholesterol, TG: Triglycerides, HbA1c: Hemoglobin A1c, FPG: Fasting plasma glucose, SBP: Systolic blood pressure, DBP: Diastolic blood pressure.

**Table 2:** Baseline characteristics of participants with or without ectopic fat obesity.

Variables	Ectopic fat obesity		*P*-value
	NO	YES	
No. of participants	12723	2741	
Sex, (men)	6175 (48.53%)	2255 (82.27%)	<0.001
Age, (years)	43.47±9.01	44.80±8.29	<0.001
BMI (kg/m^2^)	21.21 (19.54,23.02)	25.08 (23.39,27.17)	<0.001
Body weight (kg)	57.20 (50.40–64.90)	71.40 (64.80–78.70)	<0.001
WC (cm)	74.00 (68.50–80.00)	85.50 (81.00–90.50)	<0.001
Habit of exercise	2308 (18.14%)	401 (14.63%)	<0.001
Drinking status			0.035
None	9717 (76.37%)	2088 (76.18%)	
Light	1472 (11.57%)	286 (10.43%)	
Moderate	1110 (8.72%)	250 (9.12%)	
Heavy	424 (3.33%)	117 (4.27%)	
Smoking status			<0.001
Never	7805 (61.35%)	1226 (44.73%)	
Past	2226 (17.50%)	726 (26.49%)	
Current	2692 (21.16%)	789 (28.79%)	
ALT (IU/L)	15.00 (12.00–20.00)	27.00 (20.00–39.00)	<0.001
AST (IU/L)	17.00 (14.00–20.00)	21.00 (17.00–26.00)	<0.001
GGT (IU/L)	14.00 (11.00–20.00)	23.00 (17.00–35.00)	<0.001
HDL-C (mmol/L)	1.48 (1.24–1.76)	1.15 (0.99–1.34)	<0.001
TC (mmol/L)	4.99 (4.45–5.59)	5.44 (4.86–6.00)	<0.001
TG (mmol/L)	0.67 (0.46–0.97)	1.25 (0.88–1.82)	<0.001
HbA1c (%)	5.10 (4.90–5.40)	5.30 (5.10–5.50)	<0.001
FPG (mmol/L)	5.14±0.40	5.40±0.36	<0.001
SBP (mmHg)	111.50(102.00,121.50)	122.50(113.50,132.50)	<0.001
DBP (mmHg)	69.50 (63.00–76.50)	77.50 (71.00–84.50)	<0.001

Abbreviations as in Table 1.

### Association between TG and incident of ectopic fat obesity

Before establishing the logistic regression model, we performed multiple linear regression tests on all variables and assessed the collinearity between variables according to VIF (S1 Table). We eliminated three variables with multicollinearity (body weight, DBP and WC). The significant variables (*P*<0.05) in univariate analysis (S2 Table) and noncollinear variables were incorporated into the multivariate regression model. Table 3 summarizes the association between TGs and ectopic fat obesity. In the crude model, there was a positive correlation between TGs and ectopic fat obesity (OR = 4.13, 95% CI:3.85–4.44, *P*<0.0001), and there was the same positive correlation shown in the fine-tuning model (Model I: adjusted for sex, age, and BMI; OR:2.09, 95% CI:1.94–2.26, *P*<0.0001). After adjusting the full model (Model II: adjusted sex, age, ALT, AST, habit of exercise, GGT, HDL-C, TC, HbA1c, smoking status, FPG, SBP and BMI), the positive correlation between them remained (OR: 1.54, 95% CI:1.41–1.69, *P*<0.0001).

**Table 3:** Association between TGs and ectopic fat obesity in different models.

Variable	Crude Model		Model I		Model II	
	OR (95% CI)	*P*	OR (95% CI)	*P*	OR (95% CI)	*P*
TG	4.13 (3.85, 4.44)	<0.0001	2.09 (1.94, 2.26)	<0.0001	1.54 (1.41, 1.69)	<0.0001
TG						
≤1.7	Ref		Ref		Ref	
>1.7	6.94 (6.20, 7.77)	<0.0001	2.91 (2.55, 3.33)	<0.0001	1.74 (1.49, 2.03)	<0.0001

Crude model was not adjusted for other variables; Model I was adjusted for sex, age and BMI; Model II was adjusted for sex, age, ALT, AST, habit of exercise, GGT, HDL-C, TC, HbA1c, smoking status, FPG, SBP and BMI; Abbreviations: CI, confidence; OR, odds ratios; *P*,*P*-value; Ref, reference.

### Analyses of nonlinear association

**Fig 1:**
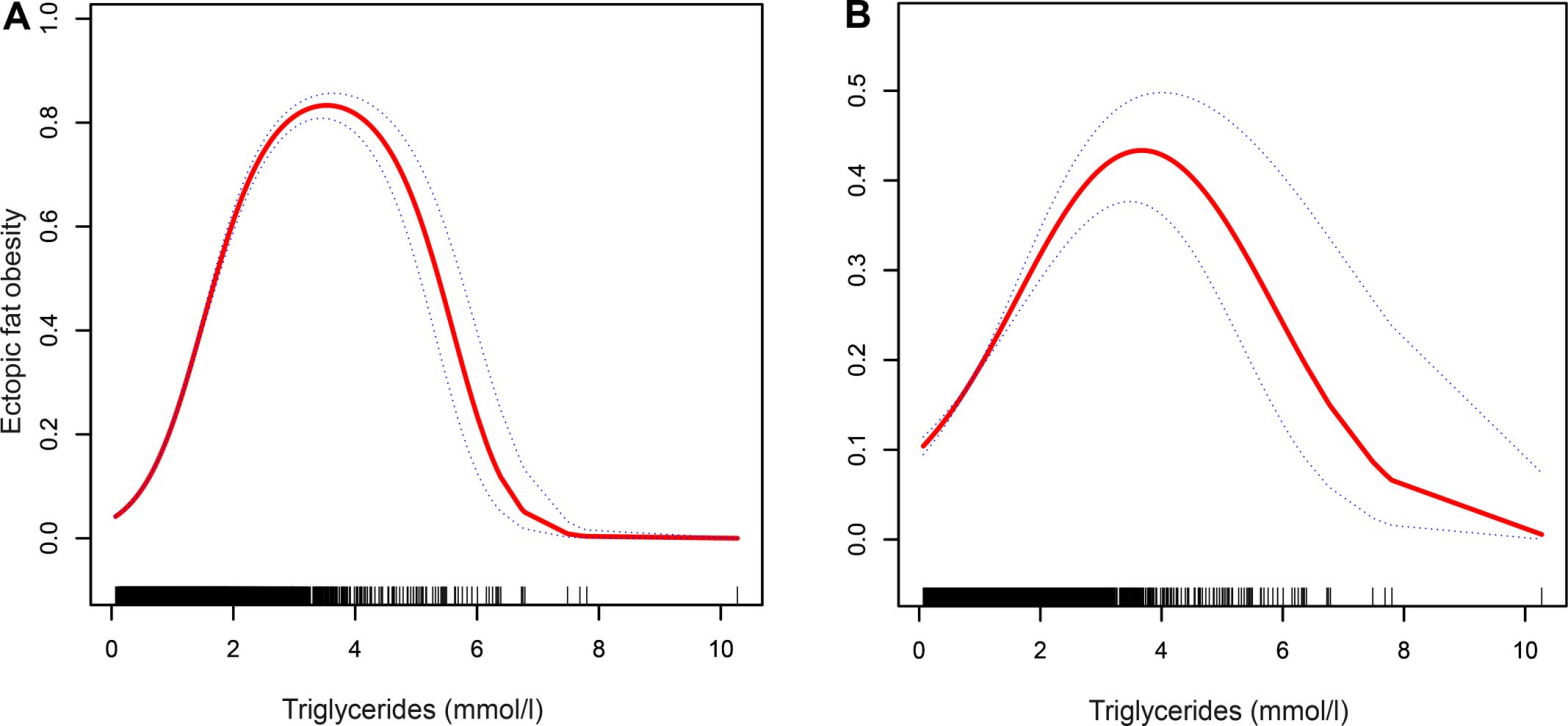
**Association between TGs and the inverted U curve of ectopic fat obesity in the unadjusted model (A) and adjusted model (B).** Model as adjusted for sex, age, ALT, AST, habit of exercise, GGT, HDL-C, TC, HbA1c, smoking status, FPG, SBP and BMI. Dotted lines represent the 95% confidence interval.

**Fig 2:**
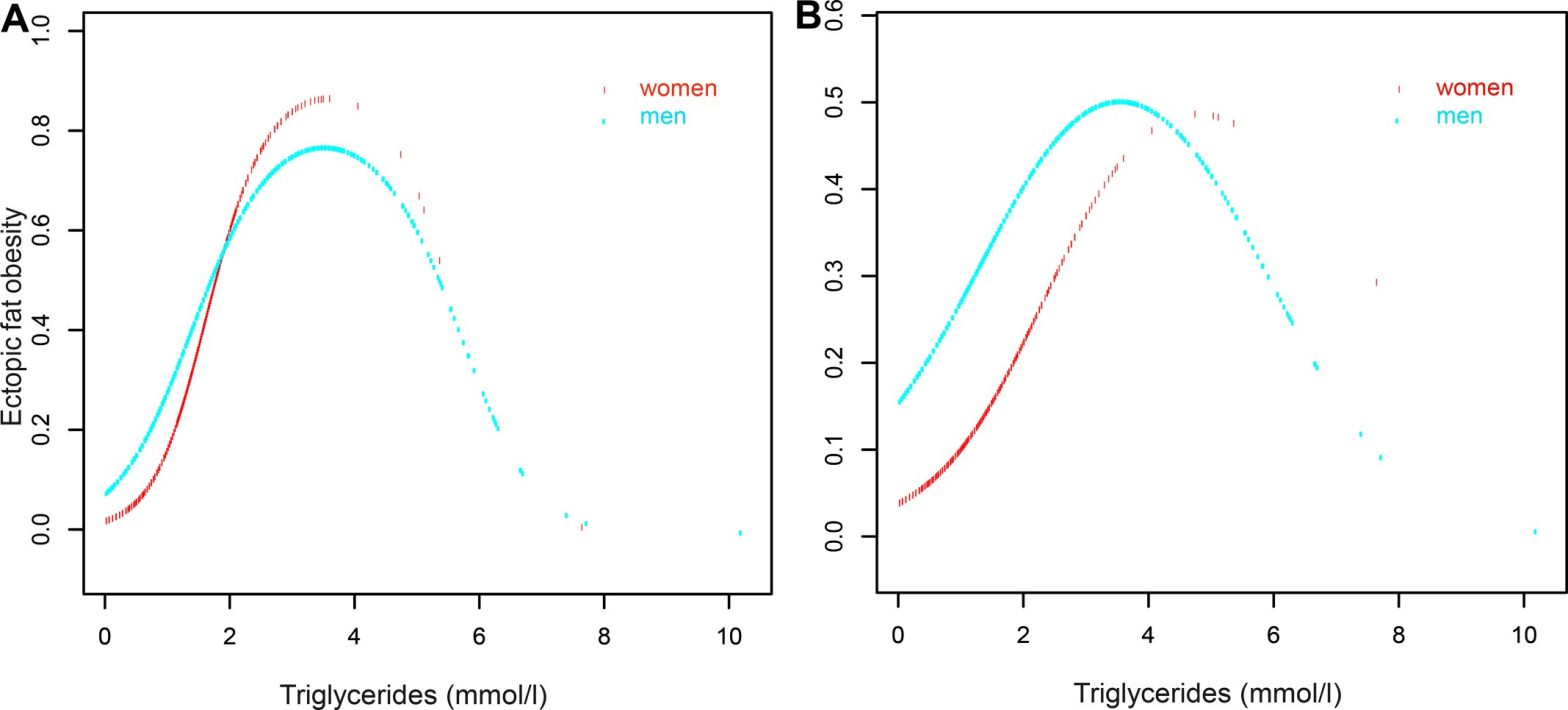
**Inverted U-shaped curve association between unadjusted (A) and adjusted (B) models for TGs and ectopic fat obesity in men and women.** Model was adjusted for age, ALT, AST, habit of exercise, GGT, HDL-C, TC, HbA1c, smoking status, FPG, SBP and BMI.

Because TG was a continuous variable in this study, we used the GAM to identify the nonlinear association between TGs and ectopic fat obesity. After adjusting other covariables, an inverted U-shaped curve association was observed between TGs and ectopic fat obesity, and the curve inflection points of TGs was in the range of 3.5–4 mmol/L as shown in Fig 1. According to gender as a stratification factor [29,30], we fitted the association between TGs and ectopic fat obesity in different genders. Fig 2 shows that there was a similar inverted U-shaped curve association between men and women, and the inverted U-shaped curve association existed after multivariable adjustment. We used Engauge Digitizer software to identify the inflection point of the curve of the association between TGs and ectopic fat obesity in the study population. Overall, the inflection point was 3.98, and the inflection point was 3.93 in men and 5.18 in women. We used a two-stage logistic regression model to calculate the saturation effect of TGs on the incidence of ectopic fat obesity according to the smoothing curve and its inflection point, and we found that there was a positive correlation between TGs and ectopic fat obesity on the left side (TG≤3.98) of the inflection point (OR:1.784, 95% CI:1.611–1.975, *P*<0.0001). On the right side (TG>3.98) of the inflection point, there was a negative correlation between TGs and ectopic fat obesity (OR:0.519, 95% CI:0.333–0.810, *P* = 0.0039) (Table 4). On the other hand, in order to further verify the stability of this curve association between different populations, we also carried out the same statistical analysis steps in the pre-set age and BMI subgroup. As expected, there is a similar inverted U curve association in most different ages and BMI stratification (S1 and S2 Figs), which further supported the stability of the inverted U curve association between TGs and ectopic fat obesity in the general population.

**Table 4:** Two-stage logistic regression model results.

	Ectopic fat obesity (OR, 95% CI)	*P*-value
Fitting model by standard linear regression	1.545 (1.413, 1.688)	<0.0001
Fitting model by two-stage linear regression		
The inflection point of TGs	3.98	
≤3.98	1.784 (1.611, 1.975)	<0.0001
>3.98	0.519 (0.333, 0.810)	0.0039

The model was adjusted for sex, age, ALT, AST, habit of exercise, GGT, HDL-C, TC, HbA1c, smoking status, FPG, SBP and BMI; Abbreviations: CI: Confidence interval; OR: Odds ratios.

### Subgroup analyses

To better understand other possible influencing factors in the risk of TGs and ectopic fat obesity, we conducted stratified analysis and interaction tests in pre-defined subgroups (Table 5); the interaction analysis detected that sex and BMI played an interactive role in the association between TGs and ectopic fat obesity (*P* for interaction <0.0167). Additionally, in the stratified analysis of sex and BMI, we observed that the risk of ectopic obesity was more greater in men (OR:2.232, 95% CI:1.787–2.787), and underweight people (BMI <18.5 kg/m^2^: OR:1.834, 95%CI:0.614–5.478).

**Table 5:** The effect size of TGs on ectopic fat obesity in prespecified and exploratory subgroups.

Characteristic	No. of participants	OR (95% CI)	*P* for interaction*
Age (years)			0.1035
18–29	416	2.308 (0.846, 6.298)	
30–39	5175	1.845 (1.566, 2.175)	
40–49	5786	1.521 (1.344, 1.722)	
50–59	3375	1.409 (1.210, 1.641)	
60–69	656	1.269 (0.965, 1.669)	
≥70	56	1.712(0.231, 12.715)	
Sex			0.0003
men	8430	2.232 (1.787, 2.787)	
women	7034	1.472 (1.343, 1.613)	
BMI (kg/m^2^)			0.0071
<18.5	1630	1.834 (0.614, 5.478)	
≥18.5, <24	10074	1.746 (1.557, 1.958)	
≥24, <28	3068	1.339 (1.188, 1.510)	
≥28	692	1.740 (1.234, 2.451)	

Note 1: The above model was adjusted for sex, age, ALT, AST, habit of exercise, GGT, HDL-C, TC, HbA1c, smoking status, FPG, SBP and BMI.

Note 2: In each case, the model was not adjusted for the stratification variable.

*Bonferroni correction for additive model; Abbreviations: CI: Confidence interval; OR: Odds ratios.

## Discussion

In this study, we identified a significant association between TGs and the incidence of ectopic fat obesity, and this association was independent of other risk factors (OR:1.54, 95% CI:1.41–1.69, *P*<0.0001). Several previous studies have reported similar results [22–24], but these studies have not determined the nonlinear association. The present study not only assessed the independent impact of TGs and ectopic fat obesity risk but also explored the nonlinear association between them. We found that there was an inverted U-shaped curve association between TGs and ectopic fat obesity even if the adjusted covariance was removed from the model or using gender as a stratification factor. This is the first time that the nonlinear association between TGs and ectopic fat obesity has been explored, and the inflection point of TGs was calculated to be 3.98. It is worth noting that this association between TGs and ectopic fat obesity had the opposite effect on the left and right sides of the inflection point. When the inflection point was ≤3.98, TGs were positively correlated with the risk of ectopic fat obesity (OR:1.784, 95% CI:1.611–1.975, *P*<0.0001), indicating that individuals with hypertriglyceridemia have the highest risk of ectopic fat obesity when TG levels range from 1.70 to 3.98. When the inflection point was >3.98, there was a negative correlation between TGs and risk of ectopic fat obesity (OR:0.519, 95% CI:0.333–0.810, *P* = 0.0039). Compared to previous studies, our researchers identified the existence of a nonlinear association and inflection points [22–24]. However, the inverted U-shaped curve association between TGs and ectopic fat obesity as well as the mechanism behind the inflection point are not clear. Based on the association between ectopic fat and metabolic dysfunction [2], this problem has important physiological and clinical significance.

In previous studies, researchers have shown that TGs represent the major form of storage and transport of fatty acids within cells and in the plasma. With regard to overnutrition and obesity, fatty acid metabolism changes, and TGs accumulate in the liver, heart or other organs, leading to ectopic fat obesity [23,31]. In a recent study, Bril F and colleagues reported the link between intrahepatic triglycerides (IHTGs) and ectopic liver fat, and they pointed out that when the accumulation of IHTGs reach approximately 6±2%, serum TGs do not continue to increase [32]. We speculate that the accumulation of IHTGs may be related to the inflection point of the inverted U curve. When the accumulation of IHTGs reaches the threshold, there is a saturation effect, which further leads to the saturation effect of TG accumulation, that is, the inflection point of TGs in the curve.

In recent years, research on ectopic fat obesity has gradually increased. Many studies have suggested that ectopic fat obesity is a significant risk factor for a variety of cardiovascular diseases and type 2 diabetes [7,15–17] and that TGs are an independent risk factor for many cardiovascular and endocrine diseases [11,19–21]. However, there is still no clear standard for the evaluation of ectopic fat obesity. In this paper, univariate analysis showed that sex, BMI, TG, HbA1c and FPG were strongly correlated with the risk of ectopic fat obesity (S2 Table). To better understand the association between TGs and the risk of ectopic fat obesity, we included the significant variables in univariate analysis (*P*<0.05) and noncollinear variables into multivariate analysis. After adjusting the covariance, TGs were confirmed to be independently related to ectopic fat obesity (OR:1.54, 95% CI:1.41–1.69, *P*<0.0001), and the risk of ectopic fat obesity in the hypertriglyceridemia group (>1.7) was 1.74 times higher than that in the normal TG group (≤1.7) (OR: 1.74, 95% CI:1.49–2.03, *P*<0.0001, *P*<0.0001 for trend). Furthermore, subgroup analysis allowed better understanding of TGs and the incidence of ectopic fat obesity in different populations. The results showed that sex and BMI played interactive roles in the association between TGs and ectopic fat obesity (*P* for interaction <0.05). According to our experience and previous literature [33,34], the treatment of hypertriglyceridemia mainly depends on drug treatment and correction of unhealthy lifestyles, especially poor diet and lack of exercise. However, there is still a lack of a standardized fat regulation program in patients with ectopic fat obesity. Based on the current research, we believe that our findings will be helpful for clinicians to evaluate the ability of patients to benefit from the current management of blood lipids. We suggest that lipid management of ectopic fat obesity should be improved and that more attention should be focused on the influence of TGs.

Although our findings are novel, there were some limitations in this observational study. First, this study adopted a cross-sectional design, preventing an explanation of the causal link between TGs and ectopic fat obesity. Second, due to the cases originating from a single medical center, the universal applicability of the sample is limited. Because this study had a large clinical sample size, however, the conclusion of the study can be considered relatively objective. Third, owing to the lack of low-density lipoprotein and other apolipoproteins in the study data, we evaluated only a few common lipoproteins, and there may be some data collection bias from uncollected lipoprotein data. However, we made strict statistical adjustments to minimize residual confounding factors. Fourth, because the previous study design excluded patients with diabetes and impaired FPG as well as patients with missing data, people with ectopic fat obesity may be underestimated given the prevalence of obesity. Fifth, because there were fewer women with higher TG levels in this study (women11.82% vs men88.18%), and it can also be seen in the curve diagram between different genders and ectopic fat obesity risk, few female's TGs was at a higher level, especially at the level higher than the inflation point, which would cause some limitations. Therefore, the evidence of this study should be cautiously generalized to the female population. Finally, although we adjusted a wide range of confounding factors, some non-measurable factors cannot be ruled out, such as dietary factors and psycho-emotional factors.

## Conclusion

Overall, our research showed that there is a significant correlation between TGs and ectopic fat obesity and that there is an inverted U-shaped curve association between them. At present, ectopic fat obesity is still a health problem that has not brought forth widespread social attention, and there is no unified standard for the treatment of regulating blood lipids. Therefore, it is of considerable significance to identify a relatively simple, stable, inexpensive and convenient index to evaluate the risk of ectopic fat obesity and guide its treatment.

## Supporting information

S1 Fig The nonlinear association of TGs with ectopic fat obesity in different age groups (adjusted for sex, ALT, AST, habit of exercise, GGT, HDL-C, TC, HbA1c, smoking status, FPG, SBP and BMI). (TIF)

S2 Fig The nonlinear association of TGs with ectopic fat obesity in different BMI groups (adjusted for sex, age, ALT, AST, habit of exercise, GGT, HDL-C, TC, HbA1c, smoking status, FPG and SBP). (TIF)

S1 Table Collinearity diagnostic steps. (DOCX)

S2 Table Results of univariate analysis. (DOCX)

S1 File (ZIP)

S2 File STROBE statement—checklist of items that should be included in reports of observational studies. (ZIP)
