# Supplementary figures and images for: The association between triglycerides and ectopic fat obesity: An inverted U-shaped curve
Source: PLoS One. 2020 Nov 30;15(11):e0243068. doi: 10.1371/journal.pone.0243068 (PMC7703893; doi:10.1371/journal.pone.0243068)

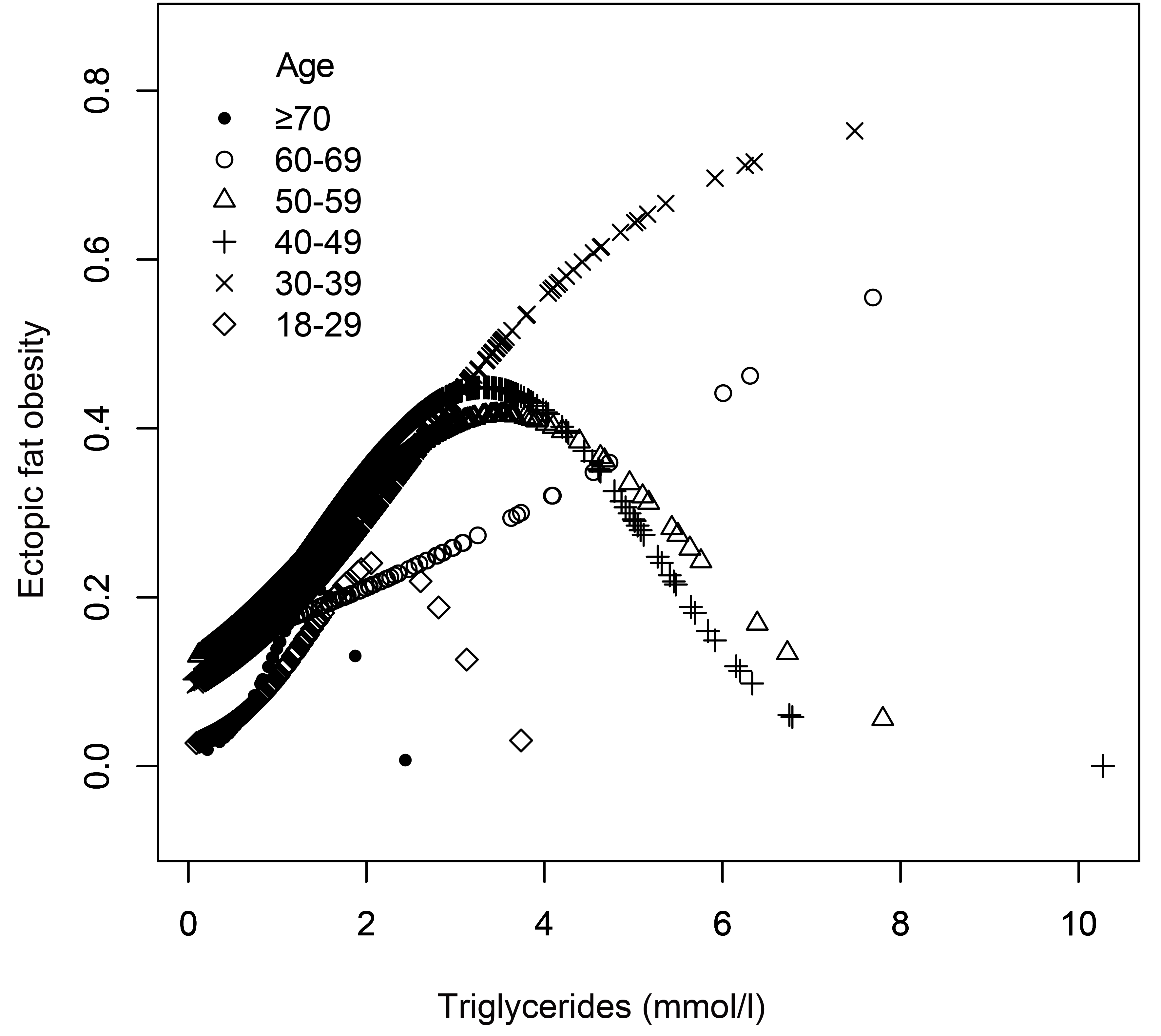

Supplement: S1 Fig — (TIF) [file pone.0243068.s001.tif]

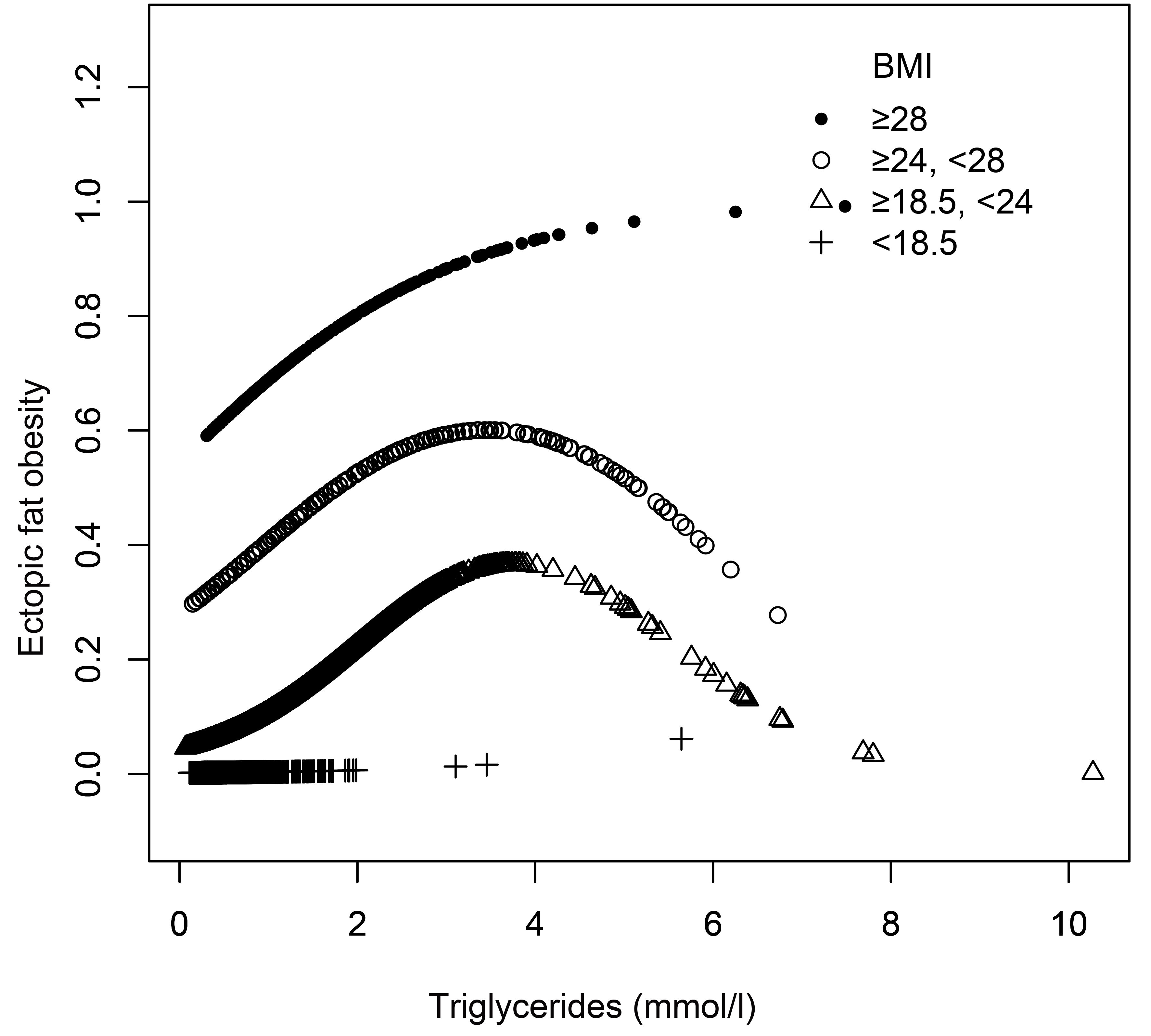

Supplement: S2 Fig — (TIF) [file pone.0243068.s002.tif]
