# Supplementary material for: The association between triglycerides and ectopic fat obesity: An inverted U-shaped curve
Source: PLoS One. 2020 Nov 30;15(11):e0243068. doi: 10.1371/journal.pone.0243068 (PMC7703893; doi:10.1371/journal.pone.0243068)
Supplement: S1 Table — (DOCX) [file pone.0243068.s003.docx]

S1 Table. Collinearity diagnostic steps.

|  | Step 1 | Step 2 | Step 3 | Step 4 |
| --- | --- | --- | --- | --- |
| Sex | 3.1 | 2.1 | 2.1 | 1.9 |
| Age | 1.4 | 1.3 | 1.2 | 1.2 |
| ALT | 3.9 | 3.9 | 3.9 | 3.9 |
| AST | 3.2 | 3.2 | 3.2 | 3.2 |
| Body weight | 9.2 | NA | NA | NA |
| Habit of exercise | 1 | 1 | 1 | 1 |
| GGT | 1.5 | 1.5 | 1.5 | 1.5 |
| HDL-C | 1.8 | 1.8 | 1.8 | 1.8 |
| TC | 1.4 | 1.4 | 1.4 | 1.4 |
| TG | 1.7 | 1.7 | 1.7 | 1.7 |
| HBA1c | 1.2 | 1.2 | 1.2 | 1.2 |
| Drinking status | 1.3 | 1.3 | 1.3 | 1.3 |
| Smoking status | 1.4 | 1.4 | 1.4 | 1.4 |
| FPG | 1.5 | 1.5 | 1.5 | 1.5 |
| SBP | 5.6 | 5.6 | 1.4 | 1.4 |
| DBP | 5.7 | 5.7 | NA | NA |
| BMI | 6.5 | 4.4 | 4.4 | 1.7 |
| WC | 5.9 | 5 | 5 | NA |

VIF: variance inflation factors. Other abbreviations as in Table 1.

VIF = 1/(1-R^2^). VIF step-by-step screening method: Calculate the VIF of each variable. If the maximum VIF value **≥**5, remove the variable with the maximum VIF value
