# Supplementary material for: The association between triglycerides and ectopic fat obesity: An inverted U-shaped curve
Source: PLoS One. 2020 Nov 30;15(11):e0243068. doi: 10.1371/journal.pone.0243068 (PMC7703893; doi:10.1371/journal.pone.0243068)
Supplement: S2 Table — (DOCX) [file pone.0243068.s004.docx]

| S2 Table. Results of univariate analysis. | | |  |
| --- | --- | --- | --- |
| Variables | OR (95%CI) | *P*-value | |
| Sex(men) | 4.920 (4.434, 5.459) | <0.00001 | |
| Age | 1.017 (1.012, 1.021) | <0.00001 | |
| BMI | 1.638 (1.607, 1.670) | <0.00001 | |
| Body weight | 1.128 (1.123, 1.134) | <0.00001 | |
| WC | 1.200 (1.192, 1.209) | <0.00001 | |
| Habit of exercise | 0.773 (0.689, 0.868) | 0.00001 | |
| Drinking status |  |  | |
| None | Ref |  | |
| Light | 0.904 (0.790, 1.035) | 0.14425 | |
| Moderate | 1.048 (0.907, 1.212) | 0.52546 | |
| Heavy | 1.284 (1.041, 1.584) | 0.01962 | |
| Smoking status |  |  | |
| Never | Ref |  | |
| Past | 2.076 (1.873, 2.302) | <0.00001 | |
| Current | 1.866 (1.689, 2.061) | <0.00001 | |
| ALT | 1.102 (1.097, 1.107) | <0.00001 | |
| AST | 1.088 (1.081, 1.095) | <0.00001 | |
| GGT | 1.032 (1.030, 1.035) | <0.00001 | |
| HDL-C | 0.058 (0.050, 0.067) | <0.00001 | |
| TC | 1.660 (1.583, 1.742) | <0.00001 | |
| TG | 4.135 (3.850, 4.440) | <0.00001 | |
| HbA1c | 4.211 (3.686, 4.809) | <0.00001 | |
| FPG | 6.591 (5.882, 7.385) | <0.00001 | |
| SBP | 1.051 (1.048, 1.054) | <0.00001 | |
| DBP | 1.076 (1.071, 1.080) | <0.00001 | |

Abbreviations: CI: confidence; OR: odds ratios; Ref: reference; other abbreviations as in Table 1.
